# The DeoR-type transcriptional regulator SugR acts as a repressor for genes encoding the phosphoenolpyruvate:sugar phosphotransferase system (PTS) in *Corynebacterium glutamicum*

**DOI:** 10.1186/1471-2199-8-104

**Published:** 2007-11-15

**Authors:** Lars Gaigalat, Jan-Philip Schlüter, Michelle Hartmann, Sascha Mormann, Andreas Tauch, Alfred Pühler, Jörn Kalinowski

**Affiliations:** 1 Institut für Genomforschung, Universität Bielefeld, D-33594 Bielefeld, Germany; 2 Lehrstuhl für Genetik, Universität Bielefeld, D-33594 Bielefeld, Germany; 3 ISAS – Institute for Analytical Sciences, Department of Metabolomics, Bunsen-Kirchhoff-Str. 11, 44139 Dortmund, Germany; 4 University of Applied Sciences Lippe and Hoexter, Life Science Technologies, Laboratory of Microbiology, Liebigstr. 87, 32657 Lemgo, Germany

## Abstract

**Background:**

The major uptake system responsible for the transport of fructose, glucose, and sucrose in *Corynebacterium glutamicum *ATCC 13032 is the phosphoenolpyruvate:sugar phosphotransferase system (PTS). The genes encoding PTS components, namely *ptsI*, *ptsH*, and *ptsF *belong to the fructose-PTS gene cluster, whereas *ptsG *and *ptsS *are located in two separate regions of the *C. glutamicum *genome. Due to the localization within and adjacent to the fructose-PTS gene cluster, two genes coding for DeoR-type transcriptional regulators, *cg2118 *and *sugR*, are putative candidates involved in the transcriptional regulation of the fructose-PTS cluster genes.

**Results:**

Four transcripts of the extended fructose-PTS gene cluster that comprise the genes *sugR-cg2116*, *ptsI*, *cg2118-fruK-ptsF*, and *ptsH*, respectively, were characterized. In addition, it was shown that transcription of the fructose-PTS gene cluster is enhanced during growth on glucose or fructose when compared to acetate. Subsequently, the two genes *sugR *and *cg2118 *encoding for DeoR-type regulators were mutated and PTS gene transcription was found to be strongly enhanced in the presence of acetate only in the *sugR *deletion mutant. The SugR regulon was further characterized by microarray hybridizations using the *sugR *mutant and its parental strain, revealing that also the PTS genes *ptsG *and *ptsS *belong to this regulon. Binding of purified SugR repressor protein to a 21 bp sequence identified the SugR binding site as an AC-rich motif. The two experimentally identified SugR binding sites in the fructose-PTS gene cluster are located within or downstream of the mapped promoters, typical for transcriptional repressors. Effector studies using electrophoretic mobility shift assays (EMSA) revealed the fructose PTS-specific metabolite fructose-1-phosphate (F-1-P) as a highly efficient, negative effector of the SugR repressor, acting in the micromolar range. Beside F-1-P, other sugar-phosphates like fructose-1,6-bisphosphate (F-1,6-P) and glucose-6-phosphate (G-6-P) also negatively affect SugR-binding, but in millimolar concentrations.

**Conclusion:**

In *C. glutamicum *ATCC 13032 the DeoR-type regulator SugR acts as a pleiotropic transcriptional repressor of all described PTS genes. Thus, in contrast to most DeoR-type repressors described, SugR is able to act also on the transcription of the distantly located genes *ptsG *and *ptsS *of *C. glutamicum*. Transcriptional repression of the fructose-PTS gene cluster is observed during growth on acetate and transcription is derepressed in the presence of the PTS sugars glucose and fructose. This derepression of the fructose-PTS gene cluster is mainly modulated by the negative effector F-1-P, but reduced sensitivity to the other effectors, F-1,6-P or G-6-P might cause differential transcriptional regulation of genes of the general part of the PTS (*ptsI, ptsH*) and associated genes encoding sugar-specific functions (*ptsF, ptsG, ptsS*).

## Background

A sugar transport system which is widespread among various microorganisms is the phosphoenolpyruvate:sugar phosphotransferase system (PTS) [1,2]. The PTS is characterized by the uptake of the carbon source, which is simultaneously phosphorylated resulting in intracellular sugar-phosphate. The transfer of the phosphate group to its substrate consists of five distinct reactions. The first step is the autophosphorylation of enzymeI, where phosphoenolpyruvate acts as the phosphate donor. Secondly, enzymeI transfers the phosphate group to the His-15 residue of the HPr protein. EnzymeI and HPr together are designated the general or central part of the PTS. HPr, in turn, transfers the phosphate group to the substrate-specific enzymesII. In general, enzymeII consists of two hydrophilic domains (IIA and IIB) and one transmembrane domain (IIC). Subsequently, the phosphate group is transferred within the enzymeII complex from domain IIA to domain IIB. The IIC-domain transports the substrate into the cell and the phosphate group is finally transfered from IIB to the substrate and the phosphorylated sugar is released into the cytoplasm [3].

The fast growing, Gram-positive bacterium *Corynebacterium glutamicum *is widely used for the fermentative production of amino acids [4] and the PTS system is the major uptake system responsible for the transport of fructose, glucose, and sucrose in this organism [5,6]. In *C. glutamicum *ATCC 13032 five genes were identified, which encode proteins with known functions in the PTS (Table 1). In detail, *ptsI *encodes the enzymeI and *ptsH *the enzyme HPr of the general part of the PTS [7]. Furthermore, *ptsG *encodes the sugar-specific enzymeII involved in the uptake of glucose, *ptsS *is involved in sucrose uptake, and finally *ptsF *is responsible for the uptake of fructose [7,8]. However, physiological results were already obtained for the function of all PTS genes with known function in *C. glutamicum *[7,9]. Additionally, for the general part of the PTS, the activities of enzymeI and HPr were demonstrated in a cell-free assay. PTS-dependent transport and the physiological function of the *ptsG *gene in the uptake of glucose were also investigated [5,10]. Genetically engineered mutations in the putative glucose- and sucrose-specific enzymesII encoded by *ptsG *and *ptsS*, revealed impaired growth on glucose and sucrose, respectively, indicating glucose transport by PtsG and sucrose transport by PtsS [8]. Inactivation of the *ptsF *gene resulted in a significantly reduced growth on fructose as a sole carbon source, therewith indicating the function of PtsF in fructose uptake [6,8]. Furthermore, PtsF-defective mutants showed a diminished growth rate compared to the wild type with sucrose as a sole carbon source, and additionally an accumulation of external fructose during exponential growth [6]. Due to the lack of a fructokinase activity in *C. glutamicum *the internally liberated fructose, resulting from the cleavage of sucrose-6-phosphate taken up by PtsS, cannot be metabolized and is exported [11,8]. Therefore, PtsF activity is also necessary to import the external fructose in order to completely metabolize sucrose. Furthermore, Moon et al. [8] identified one enzymeII encoded by *cg3365 *and *cg3366 *with unknown substrate specifity.

**Table 1:** Molecular characteristics of PTS and putative PTS associated genes of *C. glutamicum *ATCC13032

	**CDS**^1^	**NCBI No.**^2^	**gene name**	**size [aa]**	**size [kDa]**	**Annotation**
**ext.**	*cg2115*	NCgl1856	*sugR*	259	27.6	Transcriptional regulator protein, DeoR-family
	*cg2116*	NCgl1857	-	320	34.1	Putative 1-phosphofructokinase
**fructose-PTS**	*cg2117*	NCgl1858	*ptsI*	568	59.6	Phosphotransferase system (PTS), enzyme I
	*cg2118*	NCgl1859	-	264	28.0	Putative transcriptional regulator protein, DeoR-family
	*cg2119*	NCgl1860	*fruK*	330	34.0	1-phosphofructokinase
	*cg2120*	NCgl1861	*ptsF*	688	70.5	Fructose-specific PTS component, enzyme IIABC
	*cg2121*	NCgl1862	*ptsH*	89	9.1	Phosphocarrier protein HPr, PTS component
**non clustered**	*cg1537*	NCgl1305	*ptsG*	683	72.6	Glucose-specific PTS component, enzyme IIBCA
	*cg2925*	NCgl2553	*ptsS*	661	69.1	Sucrose-specific PTS component, enzyme IIBCA
	*cg3365*	NCgl2933	*-*	513	52.7	L-ascorbate type PTS component, enzyme IIC
	*cg3366*	NCgl2934	*-*	270	29.0	L-ascorbate type PTS component, enzyme IIAB

^1 ^Coding sequences (CDS) are numbered in reference to the complete genome sequence of *C. glutamicum *(GenBank accession number BX927147).

^2 ^Numbers in the genome database of the National Center for Biotechnology Information (NCBI No.).

The transcriptional regulation of genes involved in PTS transport are well studied in a variety of Gram-negative and low-G+C Gram-positive bacteria [12-15]. However, little is known about gene regulation of PTS in Corynebacteria. An HPr-kinase/phosphatase activity as well as the essentially conserved serine-residue 46 in the HPr amino acid sequence, which exerts carbon catabolite control in low-G+C Gram-positive bacteria, was not detected in *C. glutamicum *[7,16]. Additionally, genes encoding transcriptional antiterminators of the BglG-family like GlcT of *B. subtilis *(regulation of the *ptsGHI *operon) or BglG of *E. coli *(regulation of the β-glucoside PTS) are only represented by a pseudogene in the genome of *C. glutamicum *ATCC 13032 (*cg3144*'/NCgl2743') [17]. However, it has to be noted that other *C. glutamicum *isolates apparently have functional copies of antiterminator-like genes as well as additional genes for enzymes II [18]. As another transcriptional regulatory system, in the low G+C Gram-positive bacterium *Streptococcus gordonii *the DeoR-type regulator FruR was characterized, which controls fructose-PTS gene expression in dependence of fructose, sucrose or xylitol [15]. The expression of the PTS genes *ptsF*, *ptsG*, and *ptsS *in *C. glutamicum *was initially considered to be constitutive [19], but recently, *ptsG *transcription was observed to be induced by switching from biomass to L-lysine production [20]. However, a global carbon regulation in *Corynebacterium glutamicum *could not be observed until now, but candidate genes associated with the PTS were recently reviewed [21].

The aim of this study was the characterization of the transcriptional regulation of PTS genes in *C. glutamicum*. By first analysing gene transcription in the fructose-PTS gene cluster and transcriptional regulation of all known PTS genes, we found a PTS-sugar specific transcriptional regulation of all PTS genes. By deletion mutagenesis of *cg2115 *(*sugR*) and expression analysis by real-time-RT-PCR and microarray hybridization, SugR was identified as a pleiotropic transcriptional regulator of all PTS genes. Electrophoretic mobility shift assays with purified SugR protein were employed to determine the SugR binding sites and its effector metabolites.

During the preparation of this manuscript, Engels and Wendisch [22] reported the identification of the DeoR-type transcriptional regulator encoded by the gene *sugR *in *C. glutamicum*, acting as a transcriptional repressor of the *ptsG *gene. By comparing a *sugR *mutant with its parent strain by microarray hybridization, these authors found that the *ptsF *and *ptsS *genes are under the control of this regulator, which they termed SugR. The transcriptional role of SugR was extended in this work to the fructose-PTS cluster genes *ptsI*, *cg2118*, *fruK*, and *ptsH *and completes the understanding for the expression of all described PTS genes in *C. glutamicum *in general and the expression of the genes of the fructose-PTS cluster in detail. Furthermore, the effector studies in this work points to a sugar specific regulation of these genes similar to the TrmB regulator of *Thermococcus litoralis *and *Pyrococcus furiosus *[23,24].

## Results

### The extended fructose-PTS gene cluster of *C. glutamicum *contains four transcriptional units

In the genome of *C. glutamicum *ATCC 13032 [17], the locations of the five PTS genes *ptsI*, *ptsH*,*ptsF*, *ptsG*, and *ptsS *were identified. On this account, the PTS genes can be grouped into the fructose-PTS gene cluster (*ptsI*, *cg2118*, *fruK*, *ptsF*, and *ptsH*) and the distantly located glucose- and sucrose-PTS genes *ptsG *and *ptsS*, respectively, as well as the hitherto uncharacterized genes *cg3365 *and *cg3366 *(Table 1). The genes *ptsI*, *cg2118*, *fruK*, *ptsF*, and *ptsH *of the fructose-PTS cluster together with the region encoding *sugR *and *cg2116 *form the extended fructose-PTS gene cluster (Table 1).

**Figure 1:**
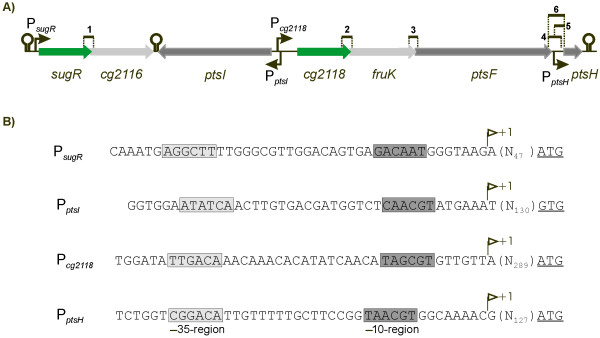
The transcriptional organization of the genes in the extended fructose-PTS cluster of *C. glutamicum*. A) The extended fructose-PTS gene cluster as well as its transcriptional elements are depicted. Gene overlapping RT-PCR products are indicated by black bars and numbered. Transcriptional terminators predicted by TransTerm [26] are denoted as stem loop structures. Positions of the transcription start sites are indicated by arrows. B) The detailed sequences for the promoters P_*sugR*_, P_*ptsI*_, P_*cg2118*_, and P_*ptsH*_. Transcription starts (+1), the distances to the translation start (in parentheses), and the start codons (underlined) are shown. The deduced-10 and -35 promoter regions are highlighted by dark and light grey boxes, respectively.

In order to analyse the transcriptional organization of the extended fructose-PTS gene cluster, mRNA transcripts overlapping adjacent coding regions were investigated by real-time RT-PCR. In these experiments, mRNAs spanning the intergenic regions between *sugR *and *cg2116*, *cg2118 *and *fruK*, *fruK *and *ptsF*, and between *ptsF *and *ptsH*, respectively, were detected in the *C. glutamicum *strain RES167 grown on glucose (data not shown). However, a non-coding region of 188 bp between the coding regions of *ptsF *and *ptsH *was observed indicating a possible promoter upstream of *ptsH*. Considering these observations and the localization of *ptsI *on the opposite strand, it is most likely, that the genes *sugR*-*cg2116 *and *cg2118*-*fruK*-*ptsF *form two operons, whereas *ptsI *is monocistronic. Although a read-through transcription from *ptsF *to *ptsH *was detected, the *ptsH *gene is probably also transcribed monocistronically. The operon predictions by the computer program VIMSS [25] and the predicted positions of transcriptional terminators 3' of *cg2116*, *ptsI *and *ptsH *determined by the computer program TransTerm [26], depicted as stem-loop structures in Fig. 1A, support this view.

Consistent with these interpretations, four transcriptional start sites were localized upstream from the genes *sugR*, *ptsI*, *cg2118*, and *ptsH*, using the rapid amplification of cDNA ends (RACE) method (Fig. 1). The deduced promoter sequences showed similarities to the sequences of the -10 (Tac/taaT) and -35 (TTTGCC/TTGGCA/TTGCCA) regions of the *C. glutamicum *consensus promoter [27]. The promoter of the *cg2118-fruK-ptsF *operon showed the highest similarity to the consensus promoter, whereas the promoters of *sugR-cg2116*, *ptsI*, and *ptsH *were less conserved, especially in the -35 region, (Fig. 1B). Furthermore, it was found that the divergent promoters P_*ptsI *_and P_*cg2118*_produce mRNAs overlapping by a complementary sequence of 14 nucleotides.

### Transcription of the fructose-PTS gene cluster is enhanced in *C. glutamicum *cultures grown on glucose or fructose when compared to cultures grown on acetate

**Figure 2:**
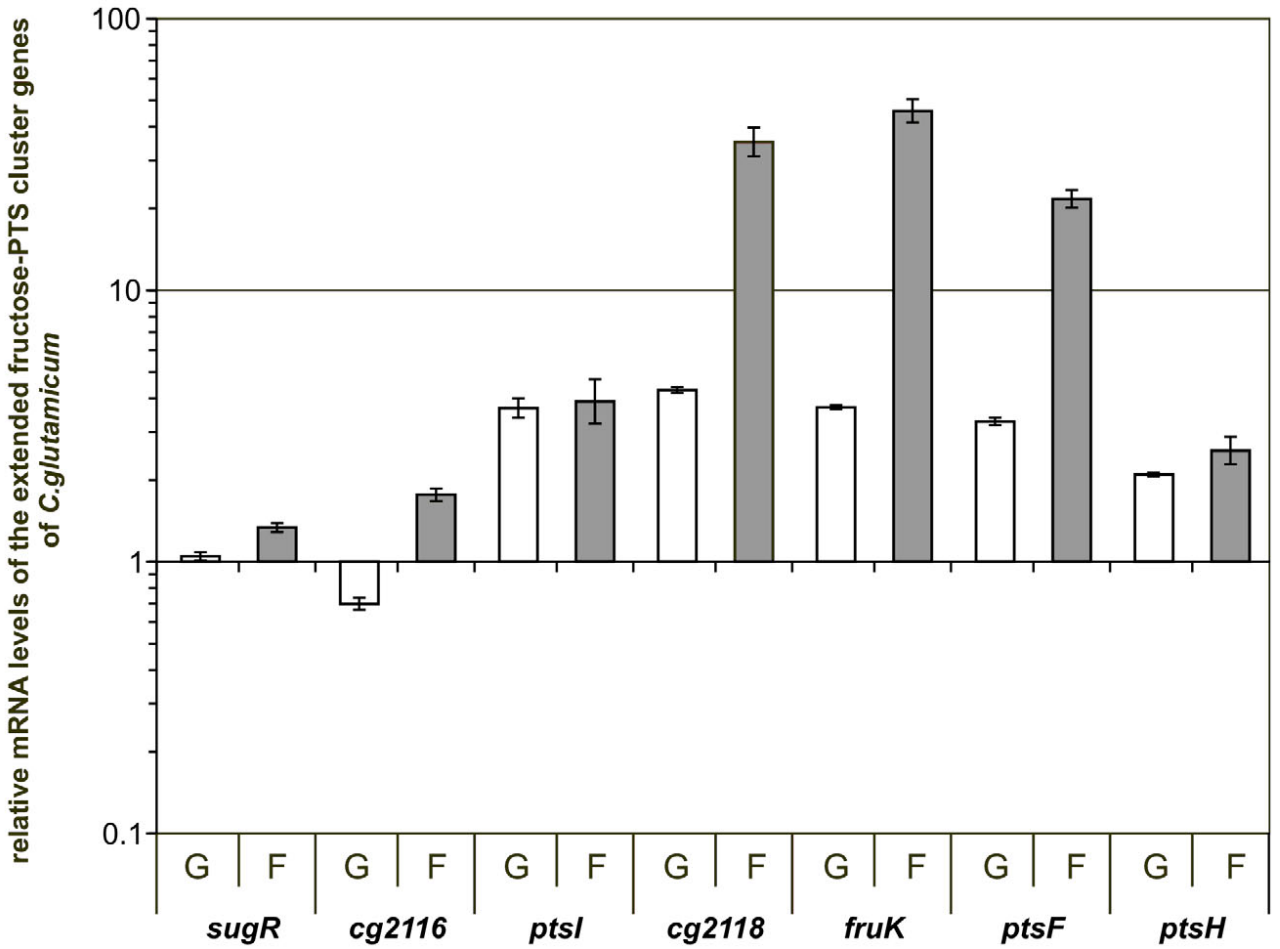
Transcriptional regulation of the extended PTS cluster genes of *C. glutamicum *cultures grown in liquid media containing glucose, fructose, or acetate. The strain RES167 was grown in liquid media containing glucose, fructose, or acetate. By RT-PCR the mRNA levels of the genes *sugR*, *cg2116*, *ptsI*, *cg2118*, *fruK*, *ptsF*, and *ptsH *of cultures grown in glucose (white bars) or fructose (grey bars) were compared to those of cultures grown in acetate containing media. Results are means of four measurements from two biological replicates. Standard deviations are indicated by error bars.

In order to investigate a carbon source-dependent expression of the genes belonging to the extended fructose-PTS gene cluster, the transcription levels were investigated by RT-PCR experiments. For this purpose, total mRNA was isolated from *C. glutamicum *RES167, grown in minimal media containing acetate, glucose or fructose as a sole carbon source. Glucose and fructose were employed as PTS sugars requiring the expression of the PTS gene cluster for their uptake, whereas acetate represents a non-PTS carbon source. The relative mRNA amounts of the genes belonging to the extended fructose-PTS cluster (*sugR *to *ptsH*) were determined using real-time RT-PCR. The values from the glucose- and fructose-grown cultures were compared to those obtained from cultures grown on acetate (Fig. 2). The mRNA levels of *sugR *and *cg2116 *showed only minor changes during growth on glucose and fructose in comparison to acetate. In contrast to this, all genes of the fructose-PTS gene cluster from *cg2117 *(*ptsI*) to *cg2120 *(*ptsH*) showed significantly higher mRNA levels during growth on glucose and fructose. The levels of the genes *ptsI *and *ptsH *did not differ between cells grown on glucose and fructose. This is in accordance with the need for expression in the presence of both sugars. In contrast to this, mRNA isolated from fructose grown cultures showed significantly higher transcription of *cg2118*, *fruK*, and *ptsF*. The different expression patterns of *cg2118*, *fruK*, and *ptsF *in cultures grown on glucose or fructose as sole carbon sources correlated with the requirement of the cell for a higher expression of the fructose-specific enzymeII (*ptsF*) and the 1-phosphofructokinase encoded by *fruK *with fructose as the sole carbon source. These results are a clear indication for a carbon source-dependent transcriptional regulation of the genes of the PTS cluster.

### The sugR gene is involved in the transcriptional regulation of the fructose-PTS cluster containing *ptsI*, *cg2118*, *fruK*, *ptsF*, and *ptsH *of *C. glutamicum*

Due to the classification of *sugR *and *cg2118 *as DeoR-type regulators, they are both interesting candidate genes for investigating their involvement in the carbon source-dependent transcriptional regulation of the fructose-PTS cluster genes. Single deletion mutants of *sugR *and *cg2118 *were constructed by gene replacement in *C. glutamicum *RES167 in order to elucidate the possible roles in this regulation (data not shown). These deletions removed 242 of 780 nucleotides from the *sugR *coding region and 674 of 795 nucleotides from the *cg2118 *coding region, rendering the respective gene non-functional and resulting in strains LG01 (RES167 Δ*sugR*) and LG02 (RES167 Δ*cg2118*), respectively.

**Figure 3:**
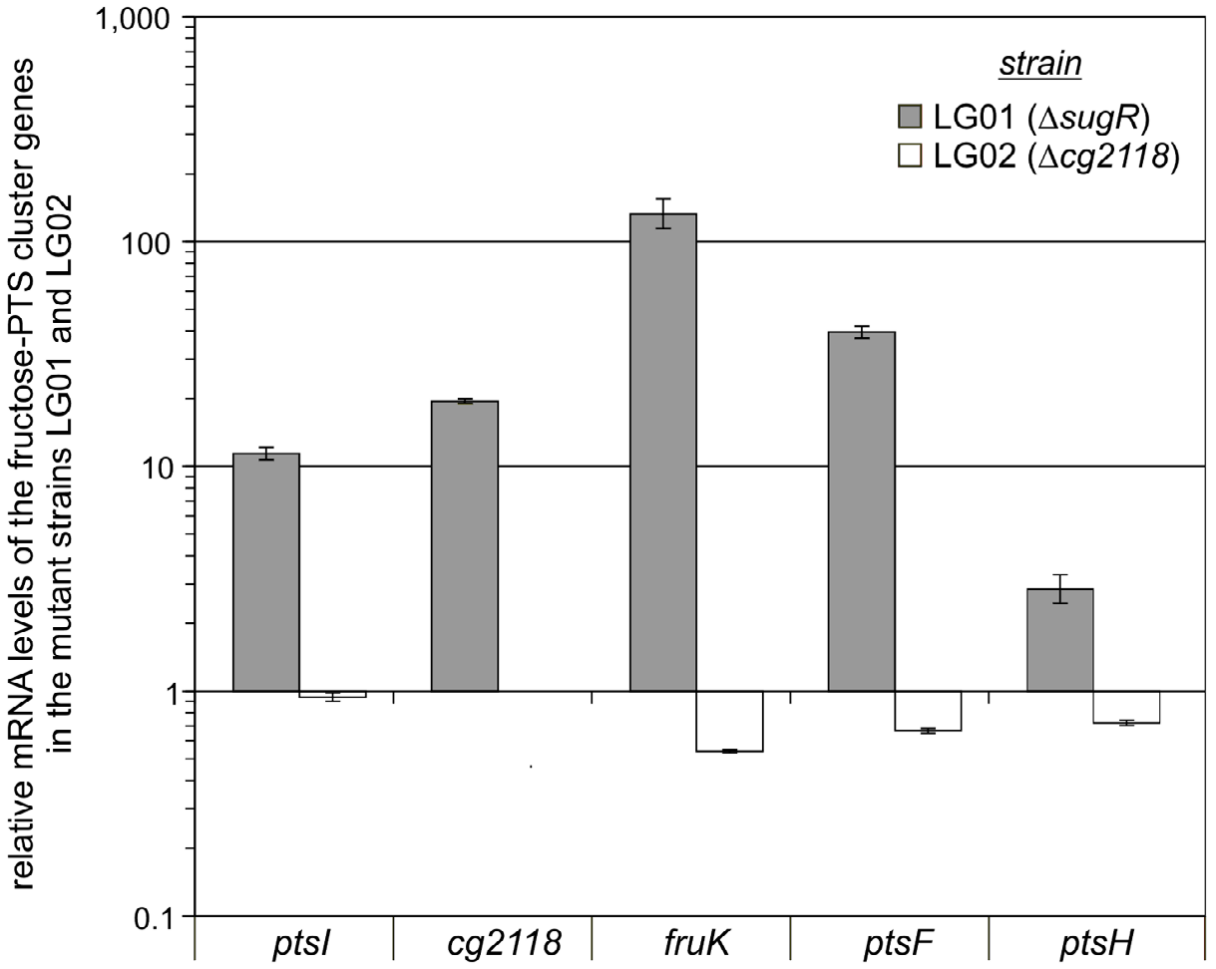
Relative gene expressions of *C. glutamicum *fructose-PTS cluster genes in dependence on the regulatory genes *sugR *and *cg2118*. The *C. glutamicum *strains LG01, LG02, and RES167 were grown in liquid media containing acetate as sole carbon source. By RT-PCR the mRNA levels of the fructose-PTS cluster genes *ptsI*, *cg2118*, *fruK*, *ptsF*, and *ptsH *of the mutant strains LG01 and LG02 were compared to those of RES167. Due to the deletion of the *cg2118 *coding region, the expression of the truncated *cg2118 *gene in the mutant LG02 could not be determined. Results are means of four measurements from two biological replicates. Standard deviations are indicated by error bars.

The possible involvement of *sugR *or *cg2118 *in transcriptional regulation of *ptsI*, the *cg2118*-*fruK*-*ptsF *operon, and *ptsH *was determined by real-time RT-PCR. For these experiments, total mRNAs isolated from RES167 and the mutant strains LG01 and LG02 were used. The cells were grown in shaking flasks in liquid minimal media with 2% acetate as sole carbon source. Acetate as the sole carbon source was chosen, because an increased transcription of the fructose-PTS cluster genes could be observed when RES167 was grown on glucose and fructose instead on acetate. Therefore, growth on acetate may cause the transcriptional repression of the fructose-PTS cluster genes. To find out whether the *sugR *or the *cg2118 *gene product is involved in the transcriptional regulation of the fructose-PTS cluster genes, the expression in the two deletion mutants LG01 and LG02 was compared to the expression in the RES167 strain when all strains were grown on acetate. The analysis revealed a strongly increased transcription of the fructose-PTS cluster genes *ptsI*, *cg2118*, *fruK*, *ptsF*, and *ptsH *in the *sugR *mutant LG01 (Fig. 3). In contrast to this, the transcription of the fructose-PTS cluster genes in the *cg2118 *mutant LG02 was only weakly influenced. It is therefore concluded, that the *sugR *gene product downregulates transcription of the fructose-PTS gene cluster during growth on acetate, whereas *cg2118 *and its gene product does not seem to be involved in the transcription of the fructose-PTS cluster genes under the applied experimental conditions.

### The *sugR *gene of *C. glutamicum *is involved in the repression of PTS gene transcription

**Figure 4:**
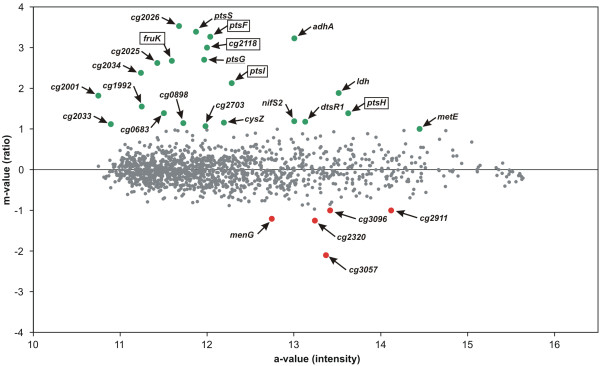
Analyses of the transcriptomes of the *C. glutamicum sugR *mutant LG01 and its parent strain RES167 by microarray hybridizations. The strains were grown in liquid minimal media containing acetate as sole carbon source. Global gene expression of LG01 was compared to that of RES167 using microarray analyses performed on two biological and two technical replicates. Genes found to be significantly differentially expressed (m-value ≥ ± 1) are indicated by green (elevated expression) or red (decreased expression) dots, respectively. Gene names belonging to the fructose-PTS gene cluster are boxed, and the non clustered PTS genes are underlined.

Comparative microarray hybridization experiments with total mRNA isolated from the *cg2115 *deletion mutant LG01 and the parental strain RES167 both grown on acetate were conducted in order to explore the complete SugR regulon. In the microarray experiments using the *C. glutamicum *whole-genome DNA microarray described previously [28], two biological replicates were hybridized, simultaneously applying label-swapping. The results of the microarray experiments comparing the *sugR *mutant and the RES167 strain revealed 23 genes with increased and five genes with decreased transcript levels in the *sugR *mutant. All genes of the fructose-PTS gene cluster were found in the group of genes with induced transcript levels (Fig. 4 and Table 2). Interestingly, the genes *ptsG *and *ptsS*, which encode the enzyme II proteins for the uptake of glucose and sucrose, respectively, were also found to have an elevated expression level. To confirm the microarray results, subsequent real-time RT-PCR measurements were conducted, which validated the enhanced transcription of *ptsG *and *ptsS *in the *sugR *mutant with ratios of 16.8 and 101.8 compared to RES167, respectively. Beside the PTS genes, the transcriptional levels of *ldh *and *adhA *encoding lactate dehydrogenase and alcohol dehydrogenase, respectively, were found to be significantly increased. Significant increases in transcript levels were also observed for *cg2025 *and *cg2026*, encoding hypothetical proteins. Besides *ldh *and *adhA*, no further genes with known function in carbon metabolism were found to be significantly induced.

**Table 2:** Differentially expressed genes deduced from the DNA microarray hybridization experiment comparing the transcriptomes of the *sugR *mutant LG01 with its parent strain RES167 during growth on acetate

**CDS**	**NCBI No.**	**Gene name**	**m-value**	**a-value**	**Annotation**
*cg2026*	-	-	3.53	11.68	Hypothetical protein
*cg2925*	NCgl2553	*ptsS*	3.39	11.87	Phosphotransferase system (PTS), sucrose-specific enzyme IIBCA component
*cg2120*	NCgl1861	*ptsF*	3.27	12.04	Phosphotransferase system (PTS), fructose-specific enzyme IIABC component
*cg3107*	NCgl2709	*adhA*	3.23	13.00	Alcohol dehydrogenase
*cg2118*	NCgl1859	-	3.00	12.00	Transcriptional regulator protein, DeoR-family
*cg1537*	NCgl1305	*ptsG*	2.70	11.97	Phosphotransferase system (PTS), glucose-specific enzyme IIBCA component
*cg2119*	NCgl1860	*fruK*	2.67	11.59	1-Phosphofructokinase
*cg2025*	-	-	2.62	11.43	Hypothetical protein
*cg2034*	NCgl1739	-	2.38	11.24	Hypothetical protein
*cg2117*	NCgl1858	*ptsI*	2.13	12.28	Phosphotransferase system (PTS), Enzyme I
*cg3219*	NCgl2810	*ldh*	1.88	13.51	L-Lactate dehydrogenase
*cg2001*	NCgl1708	-	1.82	10.75	Conserved hypothetical protein
*cg1992*	NCgl1699	-	1.55	11.25	Hypothetical protein
*cg2121*	NCgl1862	*ptsH*	1.39	13.62	Phosphotransferase system (PTS), phosphocarrier protein HPr
*cg0683*	NCgl0565	-	1.39	11.50	Putative permease
*cg2030*	NCgl1735	-	1.31	10.61	Hypothetical protein
*cg1761*	NCgl1500	*nifS2*	1.19	13.00	Cysteine desulfhydrase
*cg0812*	NCgl0678	*dtsR1*	1.18	13.13	Acetyl/propionyl-CoA carboxylase, beta chain
*cg3112*	NCgl2713	*cysZ*	1.15	12.19	Sulfate transporter
*cg0898*	NCgl0754	-	1.14	11.73	Pyridoxine biosynthesis enzyme
*cg2033*	NCgl1738	-	1.12	10.89	Putative secreted protein
*cg2703*	NCgl2373	-	1.07	11.98	ABC-type putative sugar transporter, permease subunit
*cg1290*	NCgl1094	*metE*	1.00	14.45	5-Methyltetrahydropteroyltriglutamate-homocysteine methyltransferase
*cg3096*	NCgl2698	-	-1.00	13.42	Aldehyde dehydrogenase
*cg2911*	NCgl2539	-	-1.00	14.12	ABC-type putative Mn/Zn transporter, substrate-binding lipoprotein
*cg1055*	NCgl0888	*menG*	-1.21	12.74	S-Adenosylmethionine:2-demethylmenaquinone methyltransferase
*cg2320*	NCgl2034	-	-1.25	13.24	Putative transcriptional regulator, ArsR-family
*cg3057*	NCgl2664	-	-2.10	13.37	Putative secreted protein

^1 ^Coding sequences (CDS) are numbered in reference to the complete genome sequence of *C. glutamicum *(GenBank accession number BX927147)

^2 ^Numbers in the genome database of the National Center for Biotechnology Information (NCBI No.)

Since Engels and Wendisch [22] also compared global transcript levels by microarray hybridizations in a *sugR *mutant and its parent strain, the resulting stimulon can be compared to the one obtained in this work. In the intersection of the genes showing elevated transcript levels in both experiments, only the PTS genes mentioned above are apparent. The remaining genes corresponding to each stimulon are different between both studies and might be a result of the different growth conditions used (LB versus minimal media with acetate) or might be results of an indirect effect of the derepression of PTS gene transcription.

### The SugR protein of *C. glutamicum *binds to sequences located upstream of the coding regions of *ptsI*, *cg2118*, *ptsH*, *ptsG*, and *ptsS*

To verify the direct involvement of the SugR protein in the regulation of the PTS gene cluster electrophoretic mobility shift assays (EMSA) were performed. For these experiments the SugR protein was purified after overexpression in *E. coli *as an N-terminal translational fusion with a self-cleavable intein tag using the pTYB1 vector [29]. The advantage of this system is that the native protein can be obtained without an additional attached tag sequence. The quality of the purified protein was controlled by 1-dimensional SDS-PAGE and its size was estimated to be ~28 kDa. This observation is in accordance with the annotation of the *sugR *coding region, which is predicted to encode a 27.6 kDa protein with an N-terminal DeoR-domain comprising a helix-turn-helix (HTH) motif. The identity of SugR was subsequently confirmed by peptide mass fingerprinting using MALDI-TOF mass spectrometry (data not shown).

As binding partners for SugR in the EMSA experiments, overlapping and fluorescently-labeled PCR fragments from the intergenic regions between *ptsI *and *cg2118*, as well as the regions upstream from the coding regions of *sugR, ptsH, ptsG, ptsS, adhA, cg2025*, and *cg2026 *were generated. As negative controls, internal DNA fragments of the *cg2118- *and *ptsH-*coding region were also produced by PCR and incubated with the purified protein to test for non-specific binding of the protein (data not shown).

**Figure 5:**
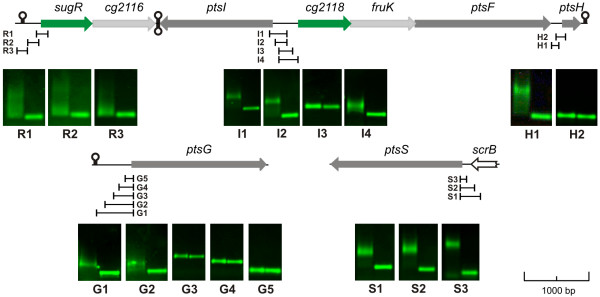
Electrophoretic mobility shift assays (EMSA) with selected upstream DNA fragments of PTS coding regions using the purified SugR protein. The physical maps of the extended fructose-PTS gene cluster as well as of the genes *ptsG *and *ptsS *are shown. Beneath the maps the fluorescently labelled PCR fragments are indicated which were used for EMSA studies. These studies were carried out with 15 pmol of purified SugR protein and 0.05 pmol of labeled PCR fragments. The results obtained for each PCR fragment are presented by agarose gel photos. In each picture, the left lane shows the shift in presence of the SugR protein, whereas the right lane shows the negative control without added SugR protein. Transcriptional terminators are denoted as stem loop structures.

For the gel retardation experiments 15 pmol of the SugR protein and 0.05 pmol of the fluorescently-labeled PCR fragments were used. It was shown that SugR caused band-shifts in experiments with DNA fragments I1, I2, and I4 located in the *ptsI*-*cg2118 *intergenic region (Fig. 5). Furthermore, binding of the purified SugR protein was observed to the DNA fragment H1 which is located upstream of the *ptsH *coding region, to the fragments G1 and G2 located upstream of the *ptsG *coding region and to the fragments S1 to S3 derived upstream of the coding region of *ptsS*.

The promoter region of *sugR *was also tested for SugR binding, but no shifts of the labeled PCR fragments were detected (Fig. 5). Furthermore, labeled PCR fragments upstream of the *cg2025*, *cg2026*, and *adhA *coding regions were tested in EMSA experiments, but no binding of SugR was observed (data not shown).

### The *C. glutamicum *SugR repressor binds to two 21 bp DNA regions interfering with *ptsI *and *cg2118-fruK-ptsF *transcription

**Figure 6:**
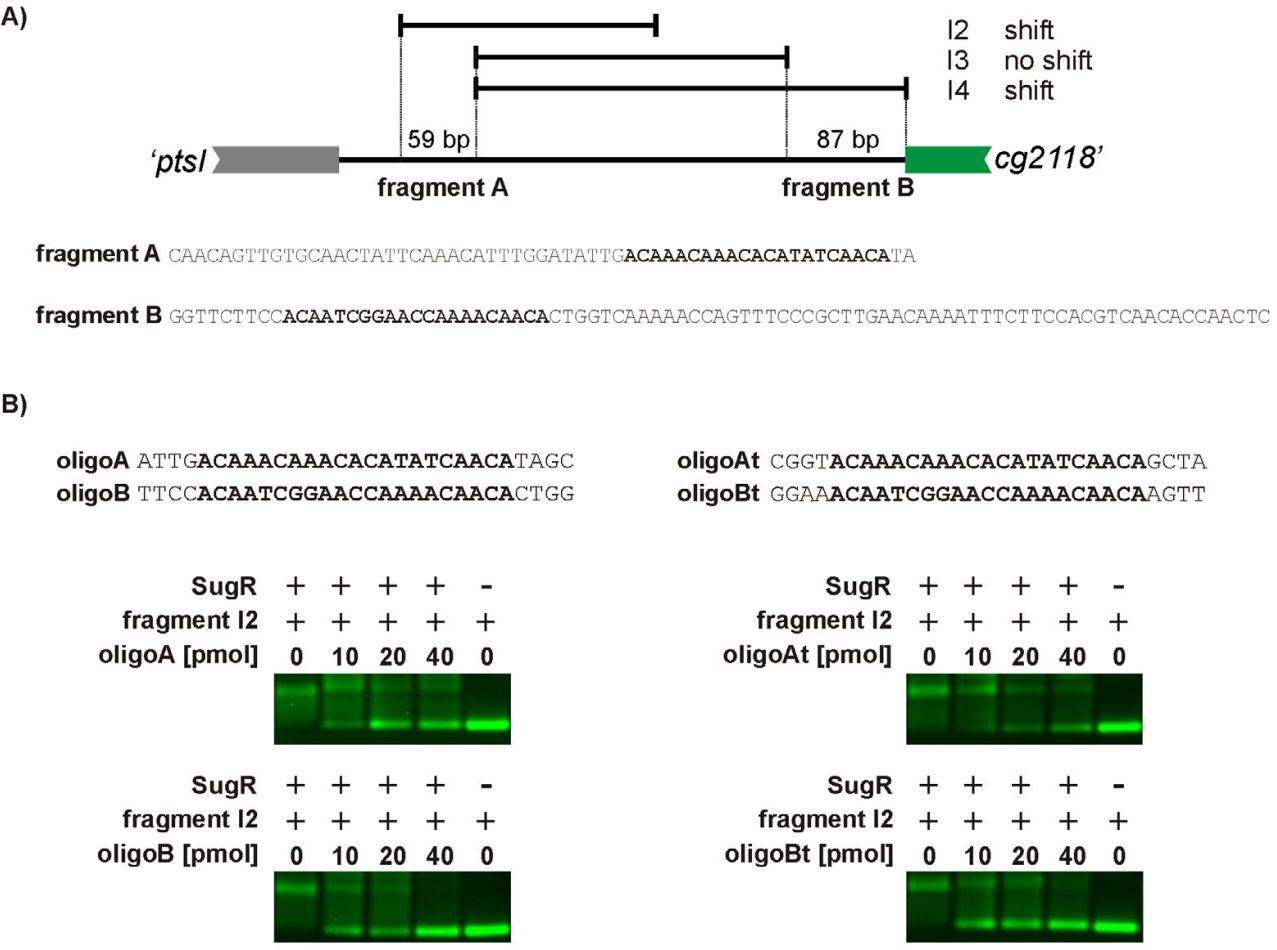
Identification of the SugR binding motifs by competitve EMSA studies. A) Detailed view of the *ptsI/cg2118 *intergenic region. For EMSA studies 15 pmol of the DNA fragments I2, I3, and I4 were used with 0.05 pmol of SugR protein. The DNA fragment I3 showed no binding of SugR, confining fragment A and fragment B carrying one SugR binding motif each. Similar sequences representing putative binding motifs in the fragments A and B are highlighted in bold letters. B) Validation of the binding motifs by competitive EMSA experiments. OligoA and oligoB represent doublestranded(ds)-oligonucleotides carrying the similar sequences of fragment A and B, respectively, with native bordering sequences. OligoAt and oligoBt represent those with modified bordering sequences. All four ds-oligonucleotides were used as competetive DNA fragments in EMSA experiments using 15 pmol purified SugR protein and 0.05 pmol of labeled DNA fragment I2. The I2/SugR-mixture was incubated with indicated amounts of the competitory (ds)-oligonucleotides. The mixture of the DNA fragment I2 and the SugR protein was used as positive control and the DNA fragment I2 alone as negative control.

As described above, SugR binding was detected to the DNA fragments I2 and I4 located in the intergenic region between *ptsI *and *cg2118*. In contrast, no binding to the DNA fragment I3 was observed. These observations helped to identify two genetic regions A and B which are proposed to carry the specific binding sites for SugR (Fig. 6A). Region A is a 59 bp long sub-region of the DNA fragment I2 and region B is a 87 bp long sub-region of I4. DNA sequence comparison of regionA to regionB revealed highly similar sequences of 21 nucleotides in length. To confirm these motifs as putative SugR binding sequences, double-stranded(ds)-oligonucleotides perfectly matching the 21 nucleotides with four flanking basepairs, representing the original genomic sequence on both sides, were used in competitive EMSA experiments and denoted as oligoA and oligoB, respectively (Fig. 6B). Interestingly, both oligonucleotides were able to displace the labeled DNA fragment I2. To further verify the binding sequence of SugR, ds-oligonucleotides with transversions of the four flanking basepairs of oligoA and oligoB, resulted in oligoAt and oligoBt, respectively, which were used in additional EMSA experiments (Fig. 6B). OligoAt and oligoBt, however, showed no differences in the competitory EMSA experiments compared to oligoA and oligoB, defining both 21 bp long binding motifs as sufficient for SugR binding. According to the localization of the two SugR binding motifs in regionA and regionB of the DNA fragments I2 and I4, respectively (Fig. 6A), these two motifs were named motifA and motifB.

**Figure 7:**
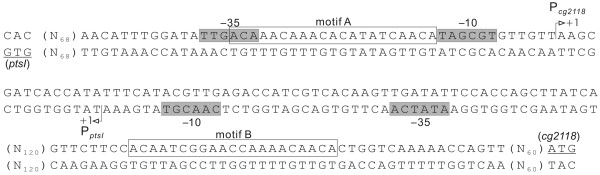
The organization of the intergenic region between the *C. glutamicum *genes *ptsI *and *cg2118 *containing the binding motifs A and B. The double stranded sequence of the intergenic region between *ptsI *and *cg2118 *is shown. The experimentally proven binding motifs A and B are also boxed. The transcriptional start sites for the two genes are indicated as P_*ptsI *_and P_*cg2118*_. The predicted -10 and -35 promoter regions are shown as dark grey boxes, respectively. The translational start codons of *ptsI *and *cg2118 *are underlined.

Subsequently, the positions of the two 21 bp SugR binding sequences motifA and motifB were mapped in the intergenic region between the coding regions of *ptsI *and *cg2118 *(Fig. 7). The two experimentally proven binding motifs in the intergenic region between *ptsI *and *cg2118 *are located within P_*cg2118*_and downstream of P_*ptsI *_(motifA), or downstream the P_*cg2118*_(motifB), respectively. Due to the divergent orientation of the coding regions *ptsI *and *cg2118 *and the overlapping transcripts from the promoters P_*cg2118*_and P_*ptsI*_, binding of SugR to motifA will interfere with transcription from both promoters, whereas the binding of SugR to motifB will only interfere with the transcription from P_*cg2118*_.

To find additional binding sites of SugR, sequence alignments of motifA and motifB to the upstream coding regions of *ptsH*, *ptsG *and *ptsS *were conducted. In these alignments, only DNA regions which showed a band-shift in the EMSA experiments were used.

**Figure 8:**
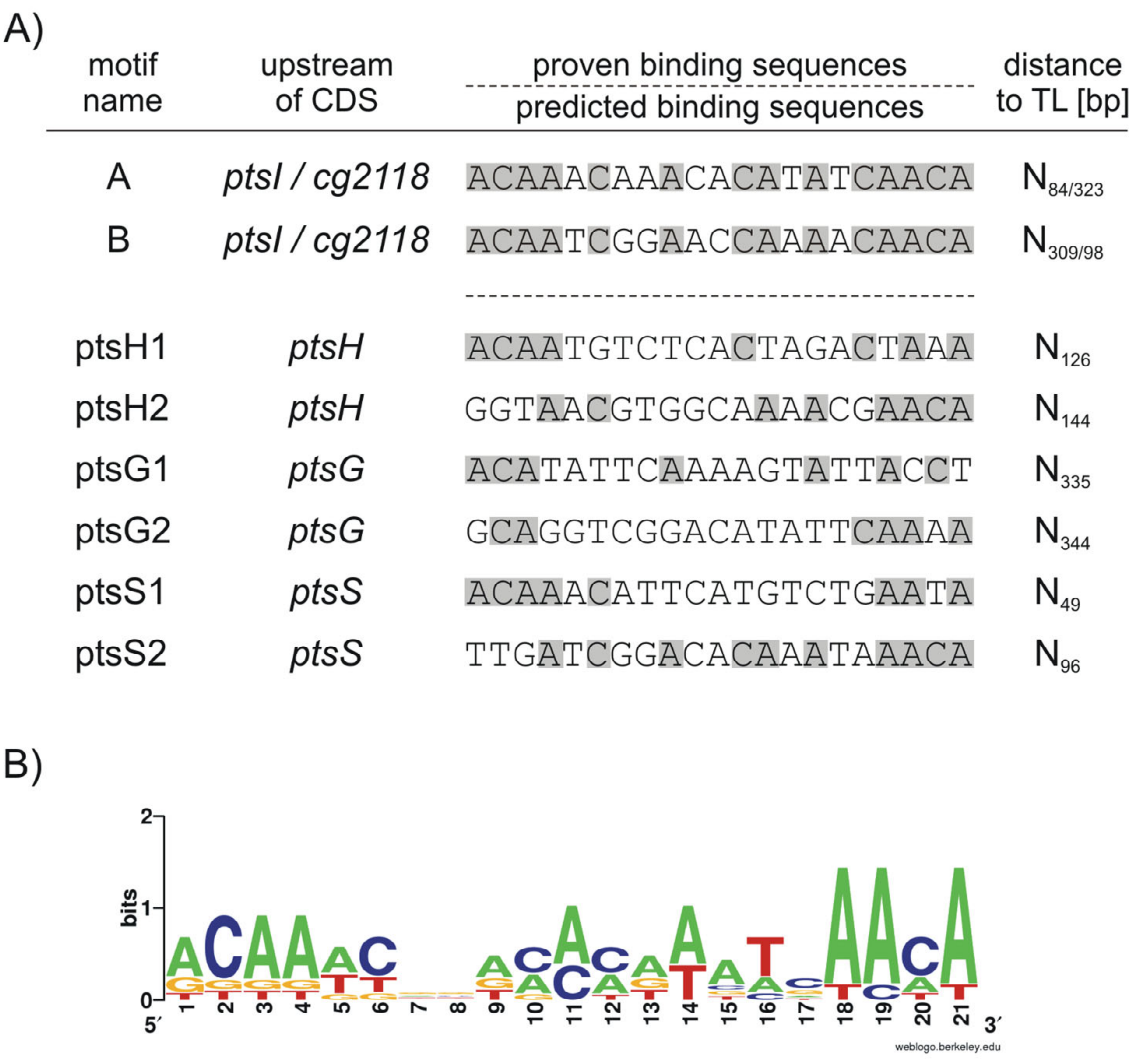
Comparison of experimentally determined and predicted SugR binding sites located upstream of the coding regions of *ptsI*, *cg2118, ptsH*, *ptsG*, and *ptsS *and construction of a consensus sequence. A) Predicted binding motifs were determined by sequence comparisons of proven motifA and motifB to the DNA fragments showing positive SugR binding in the EMSA studies. Proven and predicted motifs are separated by a horizontal, dashed line. Boxed letters in the experimentally proven motifs A and B and the putative SugR binding sequences located upstream the coding regions of *ptsH*, *ptsG*, and *ptsS *denote identical nucleotides in all sequences. Distances to the according translation starts (TL) are indicated. B) A frequency plot of the deduced consensus sequence of all motifs is constructed by means of the WebLogo tool. The overall height of each stack of letters indicates the sequence conservation at each position of the 21-bp motif, whereas the height of each symbol within the stack reflects the relative frequency of the corresponding nucleotide at that position.

By this, two putative binding motifs were found upstream of the coding regions of *ptsH *and *ptsG *and *ptsS *namely ptsH1, ptsH2 and ptsG1, ptsG2 and ptsS1, ptsS2 respectively (Fig. 8). Concluding, these experiments led to the identification of eight binding motifs. Two validated SugR binding sequences motifA and motifB located in the intergenic region of *ptsI *and *cg2118*, as well as six putative binding motifs located upstream of the coding regions of *ptsH*, *ptsG*, and *ptsS*. Sequence alignments of all eight motifs revealed that the six nucleotides ACAAAC in the 5'- and the four nucleotides AACA in the 3'-region were well conserved (Fig. 8). Additionally, a predominant occurence was observed for cytosine and adenine nucleotides at positions 9 to 17 of the consensus motif. The characteristics of the putative consensus motif were determined as an A+C-rich and non-palindromic sequence (Fig. 8).

### The effectors fructose-1-phosphate, fructose-1,6-bisphosphate, and glucose-6-phosphate influence the binding activity of the SugR repressor

In order to determine the effector substances influencing the activity of SugR, additional EMSA experiments were carried out. In these experiments, the SugR-I2 complex was incubated with fructose-1-phosphate (F-1-P) which exclusively occurs when fructose is taken up by the fructose-specific PTS. Additionally, glucose-6-phosphate (G-6-P) occurring during the transport of glucose by the glucose-specific PTS component PtsG, fructose-1,6-bisphosphate (F-1,6-P) and fructose-6-phosphate (F-6-P), originating from the consumption of different sugars, as intermediates of glycolysis were used. Furthermore, the non-phosphorylated substrates of PtsF and PtsG, fructose and glucose, respectively, as well as acetate were used.

**Figure 9:**
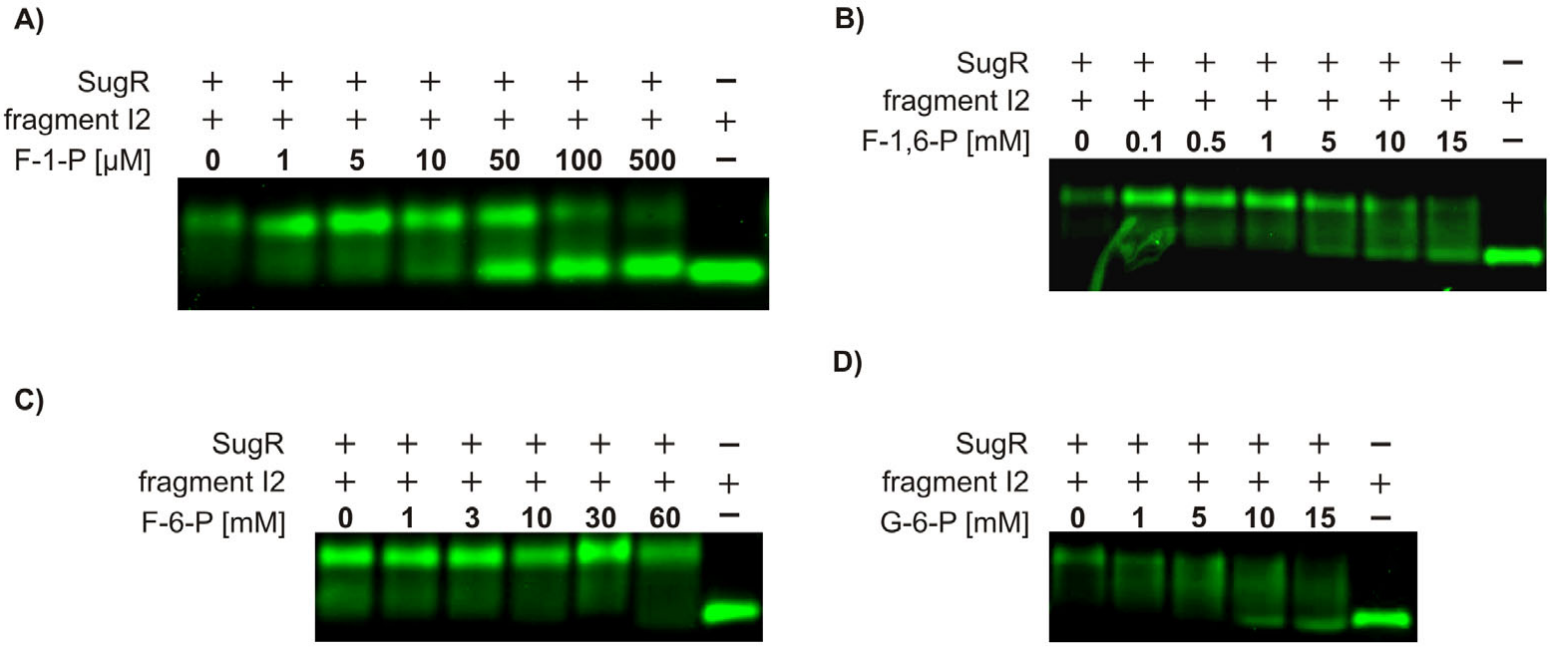
Identification of effector substances inactivating the repressor protein SugR. Electrophoretic mobility shift analysis of 15 pmol purified SugR protein complexed with 0.05 pmol of the DNA fragment I2 in the presence of different putative effectors were conducted. Effectors applied to the SugR/I2-complex at varying concentrations as described were A) fructose-1-phosphate (F-1-P), B) fructose-1,6-bisphosphate (F-1,6-P), C) fructose-6-phosphate (F-6-P), and D) glucose-6-phosphate (G-6-P)

A loss of SugR binding to the DNA fragment I2 was observed to different extents using increasing concentrations of F-1-P, F-1,6-P, and G-6-P (Fig. 9). Using F-1-P as effector substance a clear effect at concentrations equal to and exceeding 10 μM could be observed. In the case of F-1,6-P, significantly higher concentrations of the effector substance (≥ 5 mM) for resolving the SugR-I2 complex had to be applied. Additionally, G-6-P showed significant influence on the SugR-I2 complex only at concentrations above 10 mM. The binding of SugR to the DNA fragment I2 was not influenced by F-6-P concentrations up to 60 mM (Fig. 9). Additionally, no influence was observed on SugR binding using the non-phosphorylated sugars glucose and fructose as well as acetate at concentrations up to 60 mM (data not shown).

## Discussion

### The *C. glutamicum *regulatory gene *sugR *is involved in the repression of the PTS genes

In an initial study four putative transcripts were identified by RT-PCR covering *sugR *and *cg2116*, which are co-transcribed, *ptsI *and *ptsH*, which are transcribed monocistronically, and the *cg2118*-*fruK*-*ptsF *operon. Coherent with this was the experimental identification of four promoters upstream of the coding regions of *sugR*, *ptsI*, *cg2118*, and *ptsH*.

In the *C. glutamicum *strain RES167 the transcript levels of *ptsI*, the *cg2118*-*fruK*-*ptsF *operon and of *ptsH *were considerably higher on the PTS sugars glucose and fructose in comparison to the non-PTS carbon source acetate. It is interesting to note, that the transcript levels of these five genes were moderately induced in glucose-grown cells, whereas in fructose-grown cells the transcript level of the *cg2118*-*fruK*-*ptsF *operon, specifically necessary for the transport and metabolism of fructose, was much stronger induced than that of the general PTS genes *ptsI *and *ptsH*. This indicates a differential activation of transcription for the general and the fructose-specific part of the fructose-PTS gene cluster.

Microarray hybridizations comparing global transcript levels in the *C. glutamicum sugR *mutant LG01 and its parent strain, both grown on acetate, helped to confine the genes belonging to the stimulon of the LG01 mutant. For this, it was interesting to note that together with the genes of the fructose-PTS cluster, the distantly located PTS genes *ptsG *and *ptsS *were also found to be derepressed in the *sugR *mutant. These results were subsequently verified by using the more sensitive real-time RT-PCR technique verifying the expansion of the putative SugR regulon to the non-clustered PTS genes *ptsG *and *ptsS*.

In a parallel study, Engels and Wendisch [22] analyzed the same regulatory genes by constructing mutants and analysing effects on the regulation of PTS genes. They initially focussed on transcriptional regulation of *ptsG *and showed that this gene was transcriptionally regulated by the product of the *sugR *gene. By using microarray analysis to compare the global transcription levels of the *sugR *mutant to the parental strain grown on LB medium, they also ensured that *ptsS *and the genes *cg2118 *and *ptsF *are under the same transcriptional control. However, in their study the gene carrying the regulatory function was named *sugR*. By our microarray results, we were able to extend the SugR regulon by adding *fruK *and the genes encoding the general part of the PTS, *ptsI *and *ptsH*. Hence, this work shows that all functionally characterized PTS genes are under the control of the SugR regulator.

In our microarray analysis, differential regulation of the genes *cg3365 *and *cg3366*, putatively encoding a PTS component of a hitherto unknown function, was not detected. Therefore, these genes might not be a part of the SugR regulon. In contrast to this, elevated transcript levels were detected for the genes *cg2025 *and *cg2026*, encoding proteins of unknown function, and *adhA*, putatively encoding a Zn-dependent alcohol dehydrogenase. But, EMSA studies only confirmed the *in vitro *binding of SugR upstream to the according coding regions of the PTS genes. The significant expression changes of *cg2025*, *cg2026*, and *adhA *are therefore most likely indirect effects of the *sugR *mutation.

An autoregulation of SugR is unlikely, because real-time RT-PCR experiments showed only marginal deviations in the transcriptional regulation of the *sugR*-*cg2116 *operon in the *sugR *mutant in comparison with its parent strain. Coherent with this, binding of purified SugR protein upstream of the coding sequence of *sugR *was not detected.

### The DeoR-type regulator SugR acts as a repressor on transcription of all PTS genes in *C. glutamicum*

In electrophoretic mobility shift assays (EMSA), binding of the native, purified SugR protein to DNA fragments located upstream of the coding sequences of *ptsI*, *ptsH*, *ptsG*, *ptsS*, and the *cg2118*-*fruK*-*ptsF *operon was detected. This result substantiated the identification of these genes as part of the SugR regulon. Binding of SugR to its target genes occurs in the absence of effectors and transcription is negatively affected as described above. Therefore, SugR acts as repressor of transcription on PTS genes.

The results obtained in this work correlate well with the current knowledge, where DeoR-type regulators of Gram-positive bacteria often act as repressor of sugar-specific PTS genes responsible for the uptake and metabolism of sugars like fructose (*Lactococcuslactis*, [13]; *Streptococcus gordonii*, [15]), lactose (*L. lactis*, [30]; *Staphylococcus aureus*, [31]), and sorbose (*L. casei*; [32]). In *Bacillus subtilis *DeoR-type regulators are also involved in the transcriptional regulation of deoxyribonucleoside degradation [33]. The function of DeoR-type regulators was further investigated in the Gram-negative organisms *E. coli *and *Salmonella enterica *where they have transcriptional influences on non-PTS operons involved in the metabolism of deoxyribose [34,35]. A common feature of the described DeoR-type regulators is the transcriptional control restricted to neighboring genes. Solely the DeoR-type regulator DeoT of *E. coli *has potential target genes of various metabolic pathways distributed in diverse genetic loci and was designated as a master regulator [36]. In accordance to the distribution of the PTS genes in the *C. glutamicum *genome, the SugR repressor can be designated as a pleitropic regulator of the PTS genes.

### The SugR binding site comprises a 21-base pair AC-rich motif

The sequence comparisons of the SugR binding motifs led to a putative consensus sequence, which consists of two conserved non-palindromic flanking regions and an AC-rich center region. The DeoR regulators described for *E. coli *(DeoR), *B. subtilis *(DeoR), and *Salmonella enterica *(DeoQ) [34,37,35] are recognizing palindromic sequences, as well as the the DeoR-type regulator which is responsible for the transcriptional regulation of the fructose-pts gene cluster in *Streptococcus gordonii *(FruR) [15]. In addition the DeoR-type regulator of the fructose-PTS gene cluster of *Lactococcus lactis *(FruR) potentially binds to four repeating non-palindromic sequences [13]. The putative consensus sequence of the SugR binding site, however, neither shows significant similarity to the known palindromic binding sequences, nor to the non-palindromic sequences of DeoR-type repressors mentioned above.

In their parallel study, Engels and Wendisch [22] studied the binding of SugR (SugR), modified and purified by an aminoterminal decahistidine tag, to the *ptsG *upstream region. By comparing the results obtained from EMSA experiments using overlapping DNA fragments, they confined the binding site to a 23 bp sequence in which they found an 8 nt motif (5'-GTCGGACA-3') which is partly conserved in the upstream regions of *ptsS *and *cg2118*. Although the possibility remains that the SugR binding site is shorter than the 21 nucleotides as predicted here, the 8nt possibly involved in the binding of SugR as defined by Engels and Wendisch [22] is included in a putative 21 nt sequence (ptsG2) postulated in this work.

The two experimentally determined SugR binding motifsA and B are located in the intergenic region between *ptsI *and *cg2118 *and are therefore of special interest. On one hand, motifA is overlapping the promoter region of P_*cg2118*_and located downstream of P_*ptsI*_, on the other hand motifB is located downstream the promoter P_*cg2118*_. The binding within or downstream the promoter region is common to transcriptional repressors [38]. Therefore, the binding of SugR in the operator region of *ptsI *and *cg2118 *apparently results in the repression of the transcription of the divergently orientated genes. Even though the SugR binding sites upstream of the *ptsG *coding region, detected in the work of Engels et al. [22] and this work are located upstream the promoter P_*ptsG*_, a spacing of 30 bp to the -35^th ^nucleotide may be sufficient for transcriptional repression by SugR. However, the maximal repression of the *deo *operon in *E. coli *is produced by DeoR binding to three operator sequences which are overlapping and located upstream of the transcription start sites [34], resulting in cooperative binding of different regulatory sequences leading to gene repression [39]. Such behaviour is putatively not present in *C. glutamicum *since a SugR binding to DNA fragments located between transcription and translation start (fragments G3 to G5; Fig. 5) was not observed. However, further binding sites located in the coding region of *ptsG *were not ruled out by experimental approaches and therefore the repression of *ptsG *transcription by SugR remains unclear.

### Fructose-1-phosphate and other sugar phosphates act as effectors releasing SugR from its binding sites

The strong effect of F-1-P on SugR binding to the *ptsI*-*cg2118 *intergenic region is very interesting, since F-1-P is a metabolite mainly occuring during the uptake of fructose mediated by PtsF. Therefore, F-1-P is an ideal intracellular substance for sensing the presence of external fructose and efficiently induces expression of the genes encoding the general and fructose-specific parts of the PTS, *ptsI*, *ptsH*, and *ptsF*, respectively. Once external fructose is consumed, the F-1-P concentration is reduced by the activity of 1-phosphofructokinase encoded by *fruK *converting F-1-P to the less effective metabolite F-1,6-P. Although, the internal concentration of F-1-P in *C. glutamicum *grown on glucose or fructose is unknown, the internal concentrations of the effector substances G-6-P could be determined to 8 mM or 15 mM and of F-1,6-P to 23 mM or 46 mM grown on glucose or fructose, respectively. Comparing the internal amounts of G-6-P and F-1,6-P to the amounts used for the EMSA-studies suggests that SugR is negatively influenced by theses effectors *in vivo*. During the consumption of the F-1,6-P pool, the regulatory system might return to a maximal repressed state. The effector substance F-1-P was also shown to specifically affect the global regulator Cra of *E. coli *[40,41]. It is furthermore described as the main effector for the FruR repressors of the fructose operon in *Streptococcus gordonii *[15] and *Spiroplasma citri *[14].

Besides the intermediate occuring during the consumption of glucose and fructose, namely F-1,6-P, the product of glucose uptake via the PTS, namely G-6-P, is apparently able to affect SugR binding activity and to derepress transcription of the PTS genes. This would be necessary at least for the genes of the general part of the PTS (*ptsI*, *ptsH*) and for the distantly located *ptsG *gene. However, much higher concentrations of F-1,6-P or G-6-P are necessary for resolving the DNA-SugR protein complex. These results were confirmed by the RT-PCR data for the wildtype, where the transcription of the fructose-PTS cluster genes of cells grown on glucose did not reach the induction levels of cells grown on fructose, indicating higher activity of SugR on glucose then on fructose and resulting in higher induction levels of the fructose-PTS cluster genes on fructose.

As a striking difference, Engels and Wendisch [22] identified F-6-P as the only effector of SugR (SugR) when bound to the *ptsG *upstream region. F-6-P is the successive metabolic intermediate produced from G-6-P by the phosphoglucose isomerase. Therefore, F-6-P is a suitable candidate for the cell to determine whether glucose is externally available.

In the experiments performed with the *ptsI*-*cg2118 *intergenic region, no effect on SugR binding was observed for F-6-P up to concentrations exceeding 20 mM, which were applied for full resolution of the complex between SugR (SugR) and the *ptsG *upstream region in the study of Engels and Wendisch [22]. However, internal concentrations of 13 mM or 3 mM and 1 mM or 2 mM F-6-P were determined for *C. glutamicum *grown on glucose and fructose, respectively [42,19], ranging below the amounts used for the EMSA studies here.

In addition, the divergent promoters P_*ptsI *_and P_*cg2118*_are coordinately regulated as shown by the mRNA measurements in the *sugR *mutant compared to the parental strain. However, divergently transcribed genes were already described, e.g. for the *C. glutamicum *genes *aceA *and *aceB *[43]. The corresponding promoters partly overlap by their -10 region and regulation of the divergent genes *aceA *and *aceB *is obtained by binding of the regulator RamB to one binding site located in the intergenic region [44]. Hence, switching between the repressive and the activated state is coordinately triggered. Nevertheless, the *aceA *and *aceB *promoters are only overlapping by their promoter sequence. The promoters P_*ptsI *_and P_*cg2118*_are producing transcripts, which are complementary by 14 nucleotides, whereby the overlapping mRNA transcripts do not influence the regulatory coupling of the divergent genes *ptsI *and *cg2118*, but represent a hence unique promotor structure in *C. glutamicum*.

As mentioned above, binding of SugR to motifB only interferes with *cg2118*-*fruK*-*ptsF *transcription providing the opportunity for the cell to differentially regulate expression of the fructose-specific part, and the *ptsI *and *ptsH *gene of the general part of the PTS as observed for fructose and glucose grown cells. In detail, the expression of the PTS gene cluster in *C. glutamicum *grown on acetate was compared to the one on fructose and glucose, respectively, indicates that the complete gene cluster is derepressed in the presence of glucose. In the presence of fructose, however, the expression of the fructose-specific genes *fruK *and *ptsF *is significantly higher than the expression of *ptsI *and *ptsH*. Therefore, the level of the transcription from P_*cg2118*_is somehow further adjusted by the actual carbon source, whereas the transcription from P_*ptsI *_and P_*ptsH *_remains unaffected. At the moment it is not clear how this differential regulation, affecting the transcription from P_*cg2118*_, is exerted. It is possible that the SugR-DNA complexes react differentially to different effectors as it is shown for the SugR (SugR) binding site upstream of the coding region of *ptsG *[22]. This differential behaviour might be interpreted in a sense that SugR is able to form complexes with individual binding sites that have different sensitivities to sugar phosphates. A sugar specific regulation of different genes was also observed in the hyperthermophilic archaeon *Pyrococcus furiosus*, where the transcriptional regulator of the trehalose/maltose and the maltodextrin ABC transporter TrmB is inactivated by the effectors maltose and trehalose or maltotriose and sucrose, respectively [23,24]. However, the regulation of different genes by recognition of varying effectors could be an adequate explanation for the differing effectors determined by Engels [22] and this work. Furthermore, it can not be excluded that other regulators may play a role in the differential expression of the PTS gene cluster. The function of the second DeoR-type regulator gene located in the PTS gene cluster *cg2118 *in transcriptional regulation of the PTS gene cluster remained unclear. The deletion mutant of *cg2118*, however, showed no influence on transcription of the PTS genes under the tested conditions, therefore leaving the potential targets of Cg2118 unknown. However, a *sugR*/*cg2118 *double mutant (strain LG03) was constructed and investigated with RT-PCR compared to the *sugR *single mutant, but no further induction of the transcription levels were observed for the fructose PTS cluster genes when both strains were grown on fructose or acetate (data not shown). Furthermore, the Cg2118 protein was purified by the IMPACT-method described and EMSA-studies were carried out with the intergenic region of *ptsI*/*cg2118 *and the upstream region of *ptsH*. Binding of Cg2118 to the corresponding DNA-fragments was not detected (data not shown). Further microarray experiments with the deletion mutant of *cg2118 *will be a subject of further studies and may reveal the function and the regulon of Cg2118.

## Conclusion

The detailed investigation of the transcriptional regulation revealed the DeoR-type regulator SugR to be responsible for the repression of the fructose-PTS cluster genes *ptsI*, *cg2118*, *fruK*, *ptsF*, and *ptsH*, as well as of the distantly located PTS-genes *ptsG *and *ptsS*. These results were confirmed by the determined promotor and SugR-binding sequences, extending the knowledge on PTS-dependent transcriptional regulation.

The main negative effector of SugR with regard to the fructose-PTS genes (F-1-P), was different to that identified in the study concentrating on the regulation of *ptsG *(F-6-P) by Engels and Wendisch [22]. In combination, these results indicated that SugR is able to regulate genes in dependence on the presence of different effectors, reflecting a hitherto unknown regulatory mechanism in Corynebacteria.

It remains to be assessed whether the arrangement and sequence composition of the SugR-binding sites are responsible for the recognition of different effectors by SugR. Further experiments dealing with the mutational analyses of the SugR-binding sites could clarify this issue.

## Methods

### Strains and media

Bacterial strains and plasmids used in this study are listed in Table 3. All *C. glutamicum *strains were cultivated in shaking flasks at 30°C with 150 rpm in minimal medium MM1. The growth medium contained 2% (w/v) carbon source dependent on the test setup (glucose, fructose, and sodium-acetate), 1% (NH_4_)_2_SO_4_, 0.3% urea, 0.1% K_2_HPO_4_; pH 7.2. After autoclaving the following substrates were added: 50 *μ*gl^-1 ^biotin and 0.1% (v/v) trace element solution containing 2 gl^-1 ^FeSO_4 _× 7H_2_O, 2 gl^-1 ^MnSO_4 _× 1H_2_O and 50 gl^-1 ^NaCl. *E. coli *DH5α MCR and JM109 were grown in shaking flasks at 37°C with 150 rpm in Luria-Bertani (LB) medium [45]. For plasmid selection the following antibiotics were used: ampicillin (100 *μ*gml^-1 ^for *E. coli*), kanamycin (50 *μ*gml^-1 ^for *E. coli *and 25 *μ*gml^-1 ^for *C. glutamicum*), and nalidixic acid 50 *μ*gml^-1 ^for *C. glutamicum *selection.

**Table 3:** Bacterial strains and plasmids used

**Strain or plasmid**	**Relevant markers, phenotypes, and characteristics**	**Reference or origin**
*C. glutamicum *strains		
RES167	Restriction deficient mutant of *C. glutamicum *ATCC* 13032, Δ (*cglIM-cglIR-cglIIR*), Nx^r^	[47]
LG01	RES167 with *sugR *deletion, after double crossover with pLMJ1, Nx^r^	This work
LG02	RES167 with *cg2118 *deletion, after double crossover with pLMJ2, Nx^r^	This work
LG03	RES167 with *cg2118*/*sugR *double-deletion, after double crossover with pLMJ1 and pLMJ2, Nx^r^	This work
*E. coli *strains		
ER2566	F-ë-*fhuA*2 [lon] *ompT lacZ*::T7 gene1 *gal sulA*11 Δ(*mcrC-mrr*)114::IS10 R(*mcr*-73::miniTn10-TetS)2 R(*zgb*-210::Tn10)(TetS) *endA*1 [*dcm*]	New England Biolabs
JM109	*recA*1, *endA*1, *gyrA*96, *thi*, *hsdR*17, *supE*44, *relA*1, Δ(*lac-proAB*)/F' [*traD*36, *proAB*^+^, *lacI*^q^, *lacZ*ΔM15]	Takara Bio Inc.
LG21	JM109 with expression vector pLGI1 for plasmid isolation, Ap^r^	This work
LG31	ER2566 with expression vector pLGI1 for the overexpression of SugR, Ap^r^	This work
Plasmids		
pK18*mobsac*B	mobilizable *E. coli *cloning vector, allows for double crossover in *C. glutamicum*, *sacB*, *lacZ*α, Km^r^	[50]
pZErO-2	*E. coli *vector, *lac *promoter, *lacZ*α, *ccdB *lethal gene, Km^r^	Invitrogen
pTYB1	*E. coli *expression vector, C-terminal intein tag, T7 promoter, *lacI*, *rrnB *T1, Ap^r^	New England Biolabs
pLMJ1	pK18*mobsacB *containing 588 bp *sugR *deletion fragment (sugR-d1/4), obtained by *Eco*RI-*Bam*HI fusion, Km^r^	This work
pLMJ2	pK18*mobsacB *containing 1117 bp *cg2118 *deletion fragment (cg2118-d1/4), obtained by *Bgl*II-*Eco*RI fusion, Km^r^	This work
pLGI1	pTYB1 containing *sugR *(777 bp), obtained by NdeI-SapI fusion, Ap^r^	This work

* American Type Culture Collection

### PCR techniques

PCR experiments were performed by using the DNA Engine Dyad thermocycler (PTC-0220) from MJ research Inc. (Watertown, Mass.). Oligonuleotides were obtained from Operon (Qiagen, Germany). Amplification of DNA was performed with Phusion Hot Start DNA polymerase (New England Biolabs, USA), which features proof-reading activity. Initial denaturation of the template DNA was carried out at 96°C for 4 min. The PCR cycle started with denaturation for 30 s, followed by annealing at primer dependent temperature at T_m _(2AT + 4GC) [46], and extension at 72°C whereas amplification time was chosen corresponding to fragment length and speed of the polymerase. The cycle was repeated 34 times, followed by a final extension step for 7 min at 72°C. Purification of the PCR products was carried out by using a PCR Purification Spin kit (Qiagen, Hilden, Germany).

### DNA isolation, transfer and manipulation

*E. coli *DH5α MCR was used for routine recombinant DNA experiments. Plasmid DNA of *E. coli *was isolated by means of the GenElute bacterial DNA isolation kit (Sigma Aldrich, Steinheim Germany). DNA-modification, analysis by gel-electrophoresis, and ligation were applied as standard procedures [45]. Restriction endonucleases and T4 DNA ligase were obtained from Amersham-Pharmacia (Freiburg, Germany) and Roche (Mannheim, Germany). Plasmid DNA was introduced into *E. coli *and *C. glutamicum *strains by electroporation as described previously [47,48] employing the Bio-Rad Gene Pulser system (Bio-Rad, Munich, Germany).

### Construction of the *C. glutamicum *deletion mutant strains

The plasmids pLMJ1 and pLMJ2 carrying deletion fragments of the genes *sugR *and *cg2118 *were constructed using the GeneSOEing method based on the PCR-mediated recombination as described by Horton *et al*. [49] (Table 3). Artificial *Mun*I- and *Bam*HI-sites for *sugR *and *Bgl*II- and *Eco*RI-sites for *cg2118 *were added by 5'-primer extension on both ends of the deletion fragment. Subsequently the resulting fragments were cleaved by the corresponding restriction enzymes and cloned into the pK18*mobsacB *vector. The resulting plasmids pLMJ1, pLMJ2 were transferred by electroporation into *C. glutamicum *RES167 [47] and therefore used to introduce the deletions by homologous recombination into *C. glutamicum *[50]. Thus the *C. glutamicum *mutant strains LG01, LG02, and LG03 carrying deletions of the genes *sugR*, *cg2118*, and a *sugR/cg2118 *double deletion were obtained.

The *sugR*, *cg2118*, and *sugR/cg2118 *deletion mutant strains were subsequently verified by PCR using additional primers positioned outside of the deletion construct as described by Rückert *et al*. [51]).

### Genetic construction, expression and purification of heterologous expressed Intein-coupled protein SugR

The SugR protein was purified by a translational fusion to intein and subsequent affinity purification performed by the IMPACT-CN system (New England Biolabs, USA), which allows the purification of the target protein without any remaining tag by thiol-induced self-cleavage of the intein. The *sugR *expression plasmids (pLGI1) was constructed by PCR amplification of a 777 bp fragment, including the complete *sugR *gene, except the stopcodon to verify C-terminal fusion of the desired protein with a 55 kDa intein-tag of the pTYB-1 vector (New England Biolabs, USA). At the 5' end an artificial *Nde*I-site and at the 3' end of the fragment an artificial *Sap*I-site was added by 5' primer extension. As recommended by the vendor the *Nde*I-site contains an ATG sequence for translation initiation and the *Sap*I-site places the C-terminus of the target protein immediately adjacent to the intein cleavage site and results in the purification of a target protein without any extra vector-derived residues at its C-terminus. After cleavage with *Sap*I and *Nde*I the fragment was directed cloned into the expression vector pTYB1 that was cleaved in an analogous manner before. Introduction of the plasmids into *E. coli *JM109 for cloning and into *E. coli *ER2566 for expression and purification resulted in the mutant strains LG21 and LG31, respectively.

*E. coli *ER2566 containing pLGI1 used for heterologous expression of SugR was grown as a preculture o/N in 10 ml LB media with 100 μgml^-1 ^ampicillin at 37°C. The main culture with a volume of 250 ml was inoculated with a cell density of 0.1 × 10^8 ^cells × ml^-1 ^and grown to an o.D. of 0.5 to 0.8 at 37°C. The expression of the proteins was acheived by IPTG (0.5 mM) induction of the *lac*-promoter and lowering the culture temperature to 16°C in order to optimize T7 RNA-polymerase transcription. The culture was grown o/N and all cells were pelleted by 6450 × *g *for 15 min at 4°C. The pellet was then resuspended in 25 ml lysis buffer (20 mM Na_2_HPO_4_, 500 mM NaCl, 1 mM EDTA, 0.1 % TritonX-100, 20 μM PMSF, 0.1 mM TCEP, pH 8.0) and the cells were disrupted by french press procedure at medium speed with 2000 psi repeating 3 times. The debris was pelleted at 6450 × *g *for 30 min at 4°C. The supernatant containing the crude protein extract was collected for IMPACT-CN purification (New England Biolabs, USA).

A 14 ml Protino column (Macherey and Nagel, Germany) was prepared with 7 ml of chitin beads and topped with a polycarbonate filter. Subsequently the packed column was equilibrated by washing with ten column volumes of precooled (4°C) column buffer (20 mM Na_2_HPO_4_, 500 mM NaCl, 1 mM EDTA, pH 8.0). Afterwards the column is ready for sample loading at 0.5–1 mlmin^-1^. Again the column was washed with ten column volumes of column buffer which can be adjusted upto 1 M NaCl. The cleavage of the intein is induced by washing with three column volumes of cleavage buffer (20 mM Na_2_HPO_4_, 500 mM NaCl, 1 mM EDTA, 50 mM DTT, pH 8.0), sealing the column to prevent drying and subsequent incubation at 4°C for 16 h. The elution of the recombinant protein can be performed by washing the column with 1–1.5 volumes of column buffer. In this case the elution volume of 7.5 ml was applied to the column and the flowthrough was collected for the subsequent concentration step with Amicon Ultra-4 centrifugal filter device with a MW cutoff of 5 kDa (Millipore, USA) down to an approximative volume of 250 *μ*l. The concentrated elution fraction was washed twice with washing buffer (20 mM Na_2_HPO_4_, 10 mM NaCl, pH 8.0) again to a total volume of 250 *μ*l. Different aliquots of the purification procedure can be collected (e.g. crude protein extract, flow through after the binding, and washing fractions) and tested with the purified protein by 1D-SDS-PAGE.

### Separation of cytoplasmic proteins of *C. glutamicum *RES167

One-dimensional denaturing sodium dodecyl sulfate polyacrylamide gel electroporesis (1D-SDS-PAGE) to separate proteins was used as described by Laemmli *et al*. [52] with a 4% stacking gel and a 12.5% resolving gel. Samples were denaturated in the presence of 2% SDS and 4% mercaptoethanol in Tris-HCl buffer (60 mM, pH 6.8) by heating to 100°C for 5 min. Apparent molecular weights were derived from the relative mobility of standard proteins as given in the Fermentas Protein Ladder (Fermentas Life Sciences GmbH, St. Leon-Roth).

Subsequently SugR identification was obtained by peptide mass fingerprint analysis [53] utilizing the Bruker Ultraflex MALDI-TOF mass spectrometer (Bruker Daltonic, Bremen, Germany). Therefore, protein spots, which should be identified, were excised from the Coomassie stained gel and digested with a modified Trypsin enzyme (Promega, Mannheim, Germany). The protocol for tryptic digest and the settings of the MALDI-TOF-MS were described previously [52]. Peptide fingerprints thus obtained were compared with *in silico *generated tryptic fingerprints derived from the *C. glutamicum *ATCC 13032 genome data [17] by using the MASCOT software (MATRIX Science Ltd., London, UK; [54]). Best hits identified the corresponding genes of the proteins analyzed.

### Operator binding assays by electrophoretic mobility shift assay (EMSA) with purified SugR

EMSA studies were performed with a set of Cy3-labeled PCR products, which were amplified using appropiate Cy3-labeled 20 mers (Operon, Germany). Unlabeled oligonucleotides representing putative binding sequences in front of *sugR*, *ptsI*/*cg2118*, and *ptsH *were annealed with the corresponding complementary oligonucleotides under standard conditions [47]. The resulting double-stranded oligonucleotides were purified by means of Qiagen MinElute columns and used in EMSA displacement experiments.

During all EMSA studies 15 pmol of purified SugR protein was added to 4 μl reaction buffer (1 mM MgCl_2_, 0.5 mM EDTA, 0.5 mM DTT, 100 mM NaCl, 10 mM Tris, 20% glycerin, pH 7.5). Subsequently, 3 μl 87.9% glycerine, 0.05 pmol of Cy3-labeled PCR product and H_2_O was added to get a final volume of 15 μl. The assay was incubated at RT for 5 min and then separated with a 2% agarose gel prepared in gel buffer (40 mM Tris, 10 mM sodium acetate, 1 mM EDTA, pH7.8). A voltage of 14 Vcm^-1 ^was applied for 35 min. The gel was then scanned with the Typhoon 8600 Variable Mode Imager (Amersham Biosciences Europe, Germany). During effector screening studies, the purified SugR protein was incubated at RT for 10 min with acetate, glucose, fructose, fructose-1-phosphate, fructose-1,6-bisphosphate, fructose-6-phosphate, or glucose-6-phosphate prior to the addition of the Cy3-labeled PCR product. During EMSA competitory experiments, the purified SugR protein was added to reaction buffer, mixed with unlabeled competitor oligonucleotide and incubated at RT for 5 min prior to adding the labeled PCR fragments. After addition of the Cy3-labeled PCR product, the assay was incubated for additional 5 min before separating by gel electrophoresis.

### Total RNA Isolation from *C. glutamicum *cultures

Cultures were grown to the logarithmic growth phase (o.D.600 = 10) and 1 × 10^9 ^cells were harvested by centrifugation at 16,000 × *g *for 15s. The supernatant was removed by pipetting and the pellet immediately frozen in liquid nitrogen. The frozen cells were resuspended in 800 *μ*l RLT-buffer (Rneasy Mini Kit, Qiagen) and instantly disrupted by means of the Ribolyser instrument (Hybaid, Heidelberg, Germany). Disruption was performed by three time intervals of 30s at speed-level 6.5 with intermediary cooling of the probes on ice for 1 min. Preparation of total RNA from *C. glutamicum *cells was performed as described by Hüser *et al*. [55].

### Determination of transcription starts by 5'-RACE

Total RNA was isolated as described above. Primers binding downstream of the annotated translation starts of *sugR*, *ptsI*, *cg2118*, and *ptsH *were used along with 1.5 *μ*g of total RNA for cDNA synthesis. The cDNA was then modified and amplified using the 5'RACE Kit (Roche Diagnostics) according to the supplier's protocol. Resulting PCR products were ligated into the vector pCR2.1 by applying the TOPO TA cloning system and chemically competent *E. coli *TOP10 cells (Invitrogen). Sequencing of cloned RACE products was carried out by IIT Biotech (Bielefeld, Germany).

### Relative quantification of mRNA levels using real-time RT-PCR

RT-PCR primers were designed to amplify an intergenic region of the analyzed gene of about 150–200 bp length with the Primer Design 4.2 software (Sci Ed Software) and were purchased from Operon (Qiagen, Germany). The real-time RT-PCR experiment was performed using the LightCycler instrument (Roche, Germany) in combination with the QuantiTect SYBR Green RT-PCR Kit (Qiagen, Germany) mixed with the specific primers and 300 ng of sample RNA.

The RT-PCR program consists of 3 segments starting with the reverse transcription at 50°C for 20 min, followed by the initial activation of the HotStar Taq DNA polymerase at 95°C for 15 min. Thirdly the RT-PCR step was performed 55 times in cycles of: 95°C for 15s, 55°C for 30s, and 72°C for 20s. The melting curve was recorded over a range of 65 to 95°C with a heating slope of 0.1°C pers at continuous fluorescence measurement. The crossing point for each gene and condition was calculated by the data analysis method provided by the LightCycler software, using the maximum increase or acceleration of fluorescence.

### Microarray experiments and analysis

Total RNA isolated from *C. glutamicum *was also used for global transcriptional analyses. The cDNA synthesis and array hybridization were performed as described by Hüser *et al*. [55]. Data analysis was performed with the ImaGene V6.0 and EMMA V2.2 [56] software packages. Evaluation of the hybridization experiment was done as described by Rey *et al*. [57] using an *m*-value cutoff of ± 1, which corresponds to expression changes equal or greater than 2 fold. Furthermore, *m*-values were considered as significant if the Student's t-test resulted in a *p*-value ≤ 0.05. The microarrays used represent all 3002 coding regions of *C. glutamicum *RES167 as 70 mer oligonucleotides.

### Bioinformatic tools used to analyse nucleotide sequences

For interpreting the data of the *C. glutamicum *ATCC 13032 genome project the automated sequence investigation program GenDB [58] was used. Sequence comparisons were carried out by using the ClustalX software [59].

## Authors' contributions

LG carried out the experimental work and drafted the manuscript. JPS participated during the microarray analyses. MH constructed the *cg2118 *deletion mutant. SM participated during experimental work and illustration design. AT participated in supervision. AP aided in coordination and participated in supervision. JK conceived of the study and participated in coordination and supervision. All authors read and approved the final manuscript.
